# Longitudinal monitoring in Cambodia suggests higher circulation of alpha and betacoronaviruses in juvenile and immature bats of three species

**DOI:** 10.1038/s41598-021-03169-z

**Published:** 2021-12-17

**Authors:** Julien Cappelle, Neil Furey, Thavry Hoem, Tey Putita Ou, Thona Lim, Vibol Hul, Oudam Heng, Véronique Chevalier, Philippe Dussart, Veasna Duong

**Affiliations:** 1 CIRAD, UMR ASTRE (Animal, Santé, Territoires, Risques, Ecosystèmes), TA A 117/E, Campus International de Baillarguet, 34398 Montpellier CEDEX 5, France; 2 ASTRE, CIRAD, INRAE, Univ Montpellier, Montpellier, France; 3 Harrison Institute, Sevenoaks, Kent, England; 4 Fauna and Flora International, Phnom Penh, Cambodia; 5 Virology Unit, Institut Pasteur du Cambodge, Institut Pasteur International Network, Phnom Penh, Cambodia; 6 Free the Bears, Phnom Penh, Cambodia; 7 Unité des Virus Émergents (UVE: Aix-Marseille Univ-IRD 190-Inserm 1207), Marseille, France; 8 Present Address: Virology Unit, Institut Pasteur de Madagascar, Institut Pasteur International Network, Antananarivo, Madagascar

**Keywords:** Virology, Ecological epidemiology

## Abstract

Recent studies suggest that coronaviruses circulate widely in Southeast Asian bat species and that the progenitors of the SARS-Cov-2 virus could have originated in rhinolophid bats in the region. Our objective was to assess the diversity and circulation patterns of coronavirus in several bat species in Southeast Asia. We undertook monthly live-capture sessions and sampling in Cambodia over 17 months to cover all phases of the annual reproduction cycle of bats and test specifically the association between their age and CoV infection status. We additionally examined current information on the reproductive phenology of *Rhinolophus* and other bat species presently known to occur in mainland southeast China, Vietnam, Laos and Cambodia. Results from our longitudinal monitoring (573 bats belonging to 8 species) showed an overall proportion of positive PCR tests for CoV of 4.2% (24/573) in cave-dwelling bats from Kampot and 4.75% (22/463) in flying-foxes from Kandal. Phylogenetic analysis showed that the PCR amplicon sequences of CoVs (n = 46) obtained clustered in *Alphacoronavirus* and *Betacoronavirus*. Interestingly, *Hipposideros larvatus* sensu lato harbored viruses from both genera. Our results suggest an association between positive detections of coronaviruses and juvenile and immature bats in Cambodia (OR = 3.24 [1.46–7.76], p = 0.005). Since the limited data presently available from literature review indicates that reproduction is largely synchronized among rhinolophid and hipposiderid bats in our study region, particularly in its more seasonal portions (above 16° N), this may lead to seasonal patterns in CoV circulation. Overall, our study suggests that surveillance of CoV in insectivorous bat species in Southeast Asia, including SARS-CoV-related coronaviruses in rhinolophid bats, could be targeted from June to October for species exhibiting high proportions of juveniles and immatures during these months. It also highlights the need to develop long-term longitudinal surveys of bats and improve our understanding of their ecology in the region, for both biodiversity conservation and public health reasons.

## Introduction

Emerging infectious diseases are a major threat to global health as illustrated by the recent emergence of the SARS-CoV-2 in China^1^. With more than 110 million human cases as of March 2021, the resulting disease, named COVID-19, led in a few months to the lock-down of billions of people around the world, with long-term political and economic impacts ongoing and difficult to estimate. Although the proximal origin of the virus is still unknown, phylogenetic studies suggest that progenitors of the virus could have originated in rhinolophid bats in Southeast Asia^2,3^. Hence, surveillance of *Rhinolophus* species, the putative reservoir of these progenitors, could contribute to the development of prevention measures and early warning systems by identifying potentially zoonotic viruses circulating in bats and their typical circulation patterns.

The surveillance of other coronaviruses (CoVs), which are classified into four genera, *Alphacoronavirus*, *Betacoronavirus*, *Gammacoronavirus* and *Deltacoronavirus,* may also be of interest^4^. CoVs can infect a wide variety of animals, causing diseases of varying severity in their respiratory, enteric, hepatic and neurological systems. Furthermore, viruses in the *Alphacoronavirus* and *Betacoronavirus* genera have caused disease in humans^5^. CoVs have been ubiquitously detected worldwide but only *Alphacoronavirus* and *Betacoronavirus* have been reported in bats^4^. Viruses in both genera show higher detections in faecal than oral samples, suggesting bat excretions may be more important in spill-over events^5^. In southeast Asia, both genera have been detected in a wide range of bat species in Cambodia, China, Laos, the Philippines, Taiwan and Thailand^6–11^ and cross-species transmission among bat hosts appears to be common^12^. In bats, reproduction phenology is linked to climate seasonality and has been identified as an important factor influencing pathogen circulation. When reproduction is synchronized, the resulting pulse of juveniles can lead to a sharp increase of immunologically naive individuals in a bat population, amplifying circulation of the pathogen^13–15^. Indeed, the number of annual birth pulses could impact the capacity of a pathogen to persist in a population as suggested by a modelling study for filoviruses^16^. Such seasonality related to bat reproduction cycles has been suggested for coronaviruses (CoVs) in several bat species in Africa and Europe. Juveniles in particular may play an important role in the circulation of these viruses^17–21^. In Southeast Asia, a large diversity of CoVs are known to circulate in bat populations, including SARS-related CoVs in horseshoe bats (*Rhinolophus* spp*.*)^6,22^. The impact of reproductive phenology on pathogen circulation has been examined in other pathogens such as Nipah virus but limited information is available regarding the ecology of CoVs in the region’s bats^23,24^.

Our study was conducted before the COVID-19 pandemic and its main objectives were to assess the diversity of CoVs circulating in several bat species in two provinces of Cambodia and test the influence of reproductive phenology on levels of CoV circulation. We undertook monthly live-captures and sampling at two sites over 17 months between 2014 and 2016 to cover all phases of the annual reproduction cycle and specifically test the association between bat age and CoV infection status. We focused on cave-roosting bats in Kampot province and on tree-roosting fruit bats in Kandal province. Additionally, we summarize current knowledge on the reproductive phenology of *Rhinolophus* and other bat species in selected areas of Southeast Asia (mainland southeast China, Laos, Vietnam and Cambodia) to provide guidelines for targeting future surveillance and prevention efforts for CoVs that could potentially emerge in the region, including SARS-related CoVs.

## Material and methods

### Study sites

**Figure 1:**
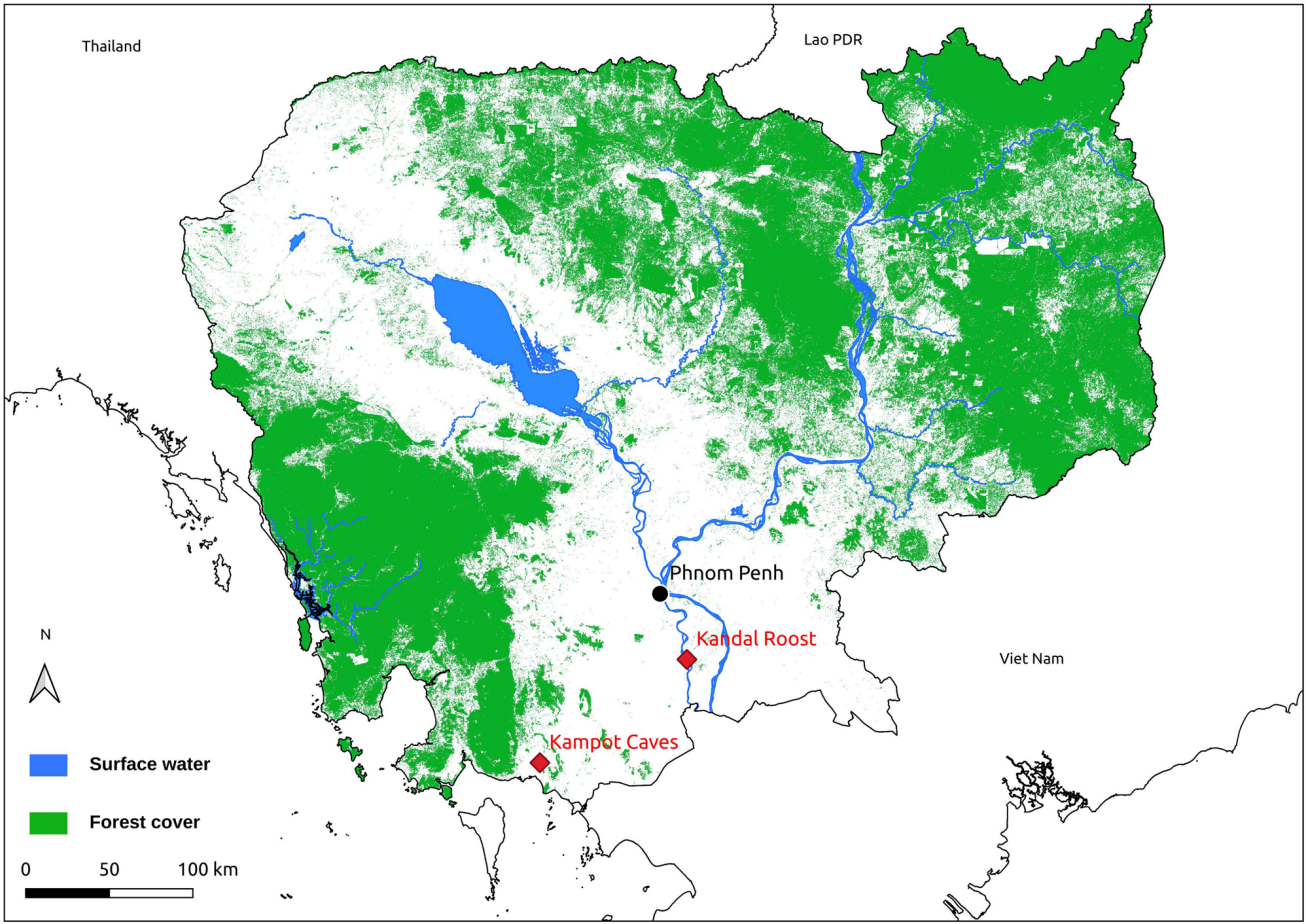
Map of Cambodia showing the two study sites (generated with QGIS 3.18, www.qgis.org).

The samples tested in this study originated from two sites in Cambodia where bats were previously captured and sampled to assess their diet and reproduction phenology (Fig. 1)^24–26^. The first site consisted of three caves (Bat Khteas, Phras Mea Kong Kea and Trai Lak) at Chhngauk hill in Kampot Province, which harbours populations of several cave-roosting bat species. Details regarding this site are provided by Thavry et al.^25^ and Lim et al.^26^. The second site was a roost of a single flying-fox species (*Pteropus lylei*) located in Kandal Province, for which further details are provided by Cappelle et al.^24^.

### Bat capture and sampling

Bat captures and sampling in Kampot Province were undertaken monthly from September 2014 to January 2016 (with the exception of October and December 2014 and January and May 2015 when the sampling team was not available). In each of the 13 sampling sessions, bats were captured using mist-nets over 3 consecutive nights (1 night per cave). Details on capture methods are provided by Thavry et al. and Lim et al.^25,26^. Bats were captured and handled in the field in accordance with guidelines approved by the American Society of Mammalogists^27^, in addition to the requirements of the statutory study permission provided by the national authority responsible for wildlife research, the Forestry Administration of the Cambodian Ministry of Agriculture, Forest and Fisheries. Ethical approval for the study was also obtained from the Forestry Administration who participated in the field investigations and oversaw all aspects of the study. In Kandal Province, the flying-fox roost site, captures and sampling were implemented monthly from August 2015 to August 2016 with two additional sessions in May 2015 and December 2016. During each session, bats were captured using mist-nets deployed in the canopy of trees surrounding the roost over 2 consecutive nights.

All bats captured were examined to determine their sex, age and reproductive status following Anthony^28^ and Racey^29^. The reproductive phenology of bat species captured at the two study sites has been described previously^24,26^. The bats were grouped according to their age and sexual maturity and status as follows: females were classified as juvenile, nulliparous, parous, pregnant and/or lactating, whereas males were classified as juvenile, immature or mature.

### Coronavirus screening

Rectal swabs were collected in the field from individual animals and placed in viral transport medium (VTM; containing tryptose phosphate Broth 2.95%, 145 mM of NaCl, 5% gelatin, 54 mM Amphotericin B, 106 U of penicillin-streptomycine per liter, 80 mg of gentamycine per liter [Sigma-Aldrich, Steinheim, Germany]) before immediate storage in liquid nitrogen for transport to the Institut Pasteur du Cambodge where they were stored at − 80 °C. In the laboratory, viral RNA was extracted using Zymo Research Direct-zol RNA MiniPrep kit (Zymo Research, Cat # R2050, CA, USA) according to the manufacturer’s instructions. The RNAs were screened with primary protocol by pan-coronaviridae RT-PCR targeting RdRp gene (adapted from Watanabe et al*.* (Watanabe-primers)^30^ and suspected positive samples were confirmed with secondary RT-PCR adopted from Quan, et al. (Quan-primers)^31^. Reverse transcription (RT) was performed using SuperScript III First-Strand Synthesis Super-Mix (Invitrogen, San Diego, CA) according to the manufacturer’s instructions.

The primary protocol PCR mixture (final volume: 25 μl) contained 2 μl of cDNA, 1 X PCR buffer, 0.20 mM (each) deoxynucleoside triphosphates (dNTPs), 1.5 mM of MgCl2, 0.2 μM of each primer (Appendix Table 1), and 2 U of Invitrogen Platinum TAQ DNA polymerase kit (Invitrogen, Carlsbad, CA, USA). The PCR mixture was incubated at 95 °C for 2 min, followed by 35 cycles of 94 °C for 30 s, 50 °C for 30 s and 72 °C for 30 s and finished with 72 °C for 5 min. The secondary protocol PCR mixture (final volume: 25 μl) contained 2 μl of cDNA, 1 X PCR buffer, 0.20 mM (each) deoxynucleoside triphosphates (dNTPs), 2 mM of MgCl2, 0.2 μM of each primer (Appendix Table 1), and 2 U of Invitrogen Platinum TAQ DNA polymerase kit (Invitrogen, Carlsbad, CA, USA). The PCR mixture was incubated at 95 °C for 5 min, followed by 15 cycles of 95 °C for 30 s, 65 °C for 30 s and 72 °C for 45 s, then 35 cycles of 94 °C for 30 s, 50 °C for 30 s and 72 °C for 45 s and finished with 72 °C for 7 min. Both nested PCR amplifications using round 2 forward and reverse primers (Appendix Table 1) were performed on 1 μl of the primary PCR products, using the same 1st round amplification conditions of each protocol. The amplification of sequences specific to CoVs was attested by the visualization of a 440 bp and a 434 bp fragments for modified Watanabe-primers and a 520 bp and a 328 bp for Quan-primers PCRs after the first and second PCR round, respectively. To limit the risk of contamination, RNA extraction, RT and PCR, nested-PCR and gel electrophoresis were carried out in separate rooms. In addition, negative controls (water) were included in each run of the nested-RT-PCR assay and results were validated only if these controls tested negative while the positive controls had to be positive. Positive PCR products were sequenced in both directions by direct Sanger sequencing at a commercial facility (Macrogen, Inc., Seoul, Republic of Korea).

### Sequence analysis

Sequences were assembled and analyzed using CLC Genomics Workbench version 3.6.1 and BioEdit, version 7.0.9.1. Sequences were aligned with a representative set of CoV sequences including previously published CoVs in Cambodia from GenBank (Appendix Table 2) using MEGA version 6.06^32^. Phylogenetic trees based on RNA sequences were constructed using the Maximum Likelihood method with the GTR + G4 + I model and bootstrap values were calculated after 1000 replicates. The best evolutionary model, phylogenetic and molecular evolutionary analyses were conducted using MEGA version 6.06^32^.

### Drivers of coronavirus positivity

We employed a generalized linear mixed model (GLMM) to test the impact of several drivers on the probability of a bat being positive by PCR for CoV infection (lme4 package in R software^33^). The result of the PCR test was the response variable with a binomial distribution. We used species, sex and age (classified in two categories, “mature adults” and “juveniles and immatures”) as explanatory variables with a fixed effect. To account for clustered samples collected during the same sampling session at the same site, we included session identification code as a random effect to control for repeated measures from the same trapping session.

### Diversity and reproductive phenology of ***Rhinolophus*** and other bat species in mainland southeast China, Laos, Vietnam and Cambodia

Because bats within the *Rhinolophus* genus are suspected to be the reservoir of the progenitors of both SARS-CoV-1 and SARS-CoV-2, we reviewed the literature to summarize current knowledge of their diversity and reproductive phenology in mainland southeast China (defined here as east of the Mekong River and south of the Yangtze River), Vietnam, Laos and Cambodia. Information on species occurrence was largely based on Burgin^34^ which is the most recent and comprehensive treatment for the genus within the region, supplemented by verified records of previously undocumented rhinolophid species in Cambodia (Supplementary Materials). As Google Scholar has been found to provide superior coverage compared to major bibliographic databases^35^, this was employed to assess the literature on reproductive phenology for our region between 2000 and 2021. The following keywords were used as search criteria: “Rhinolophus” AND “Reproductive phenology” AND “China” OR “Laos” OR “Vietnam” OR “Cambodia”. Because information on bat reproductive phenology is rarely reported for our region, we also incorporated datasets from a variety of unpublished field studies. Since climate patterns have a major bearing on the reproductive phenology of insectivorous bats^36^, we divided our region into two zones representing the two major climatic regimes present: (1) subtropics and seasonal tropics above 16° N in the north (southeast China, northern Vietnam and northern Laos) where temperatures and rainfall are noticeably seasonal (for example, in Bac Kan province [Vietnam] near the Vietnam-China border at 22° N, average monthly temperatures typically range between 12.3 and 27.5 °C and a wet summer season occurs from May to September, when ca. 75% of annual rainfall occurs^37^), and (2) increasingly tropical below 16° N (southern Vietnam, southern Laos and Cambodia) where seasonal fluctuations in temperature progressively lessen and a wet season also occurs^38^ (for instance, in Tay Ninh province [Vietnam] near the Vietnam-Cambodia border at 11° N, average monthly temperatures typically range between 25.2 and 28.9 °C, whereas ca. 85% of annual rainfall occurs from May to October^39^).

The same process was repeated for genera that tested positive for coronavirus infections in our study (*Hipposideros*, *Eonycteris* and *Pteropus*), whereby their genus names were used instead of “Rhinolophus” as keywords in search criteria during their literature reviews (all other criteria remaining unchanged). As with the review for rhinolophid bats, datasets from unpublished field studies were incorporated in each instance.

## Results

### Coronavirus screening

A total of 573 bats belonging to 8 species were captured and sampled at the first site in Kampot province and 463 *Pteropus lylei* were captured and sampled at the second site in Kandal province (Appendix Table 3). Among the samples tested, 46 different rectal swabs tested positive for CoV, including 45 with Watanabe-primers and 21 with Quan-primers (meaning that 20 samples tested positive with both sets of primers and only 1 sample tested positive with Quan-primers only). The overall proportion of positive PCR tests for CoV was 4.2% (24/573) in the cave-dwelling bats from Kampot and 4.75% (22/463) in the flying-foxes from Kandal. Three species were dominant in the caves in Kampot: *Hipposideros larvatus* sensu lato (s.l.) with 277 individuals (11 PCR positive, 10 with modified Watanabe-primers and 1 with Quan-primers, 4.0%), *Eonycteris spelaea* with 128 individuals (13 PCR positive with modified Watanabe-primers, 10.2%) and *Taphozous melanopogon* with 110 individuals (no PCR positive). Only 22 individuals from a single *Rhinolophus* species were captured (*R. malayanus*, no PCR positive). Another 36 samples were collected from 4 other bat species (22 *Hipposideros armiger*, 8 *Lyroderma lyra*, 5 *Megaderma spasma* and 1 *Cynopterus* sp., no PCR positive).

**Figure 2:**
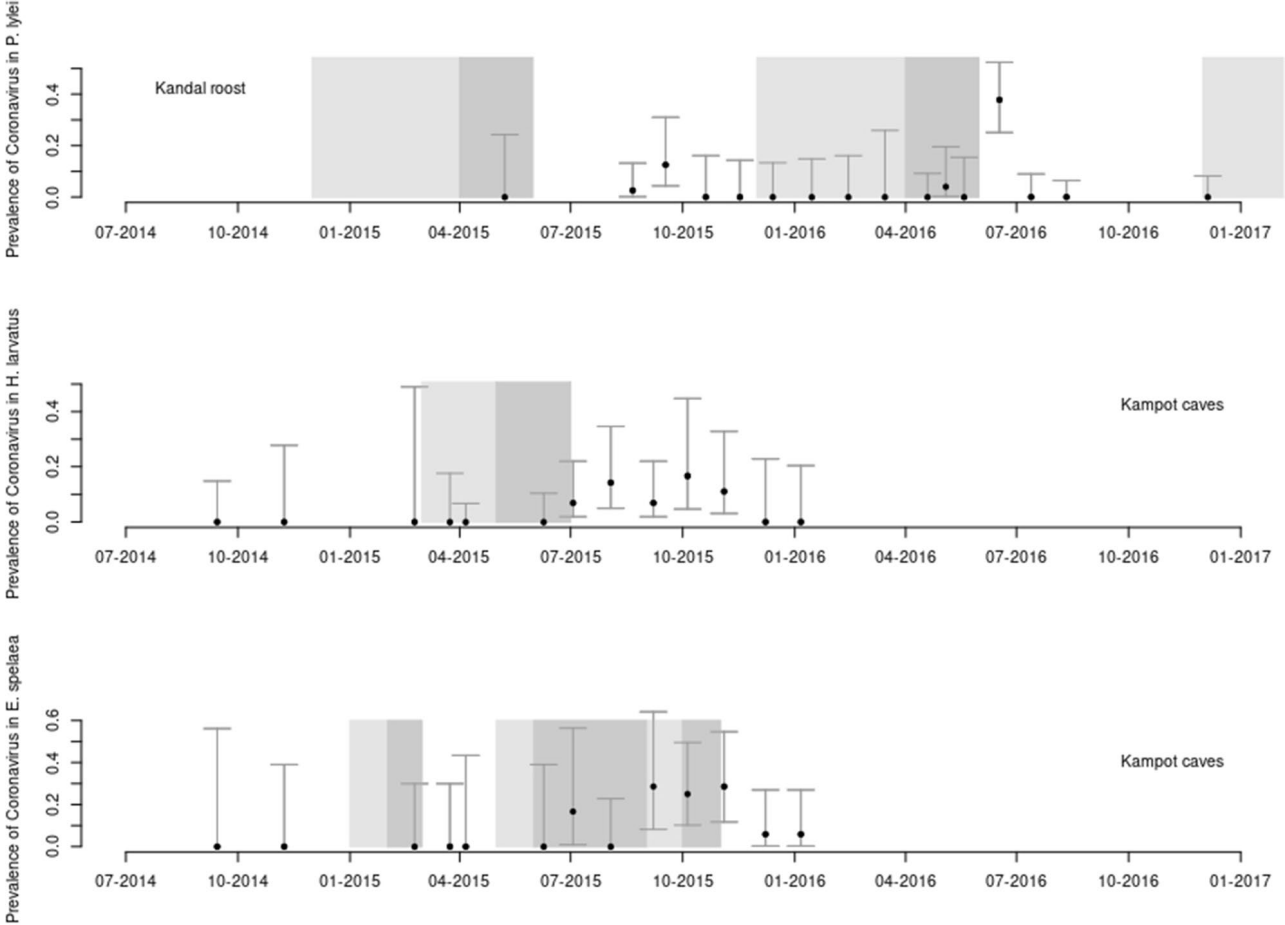
Results of the longitudinal sampling of three bat species for coronaviruses. The first graph shows estimation of the coronavirus prevalence (with CI95) for *Pteropus lylei* at the second study site in Kandal province. The second and third graph show the same for *Hipposideros larvatus s.l.* and *Eonycteris spelaea* (respectively) at the first study site in Kampot province. The light grey areas show periods when pregnant females were captured, whereas dark grey areas show periods when lactating females were captured.

Figure 2 shows monthly variation in coronavirus prevalence in the three most-captured species with at least one positive individual (detailed results are available in Appendix Tables 3, 4). For both *H. larvatus* s.l. and *E. spelaea*, prevalence was higher between July and November, i.e. 3–7 months after the birthing period. For both species, sexually immature individuals were predominant among the PCR-positive individuals: 76.9% (10/13) for *E. spelaea* and 72.7% (8/11) for *H. larvatus* s.l. For *P. lylei*, a peak in prevalence was observed in June 2016 with 17 positive individuals out of 45 sampled (37.8%), including 14 juveniles (82.4%), 2 other non-sexually mature individuals and only one sexually mature bat. An increase in prevalence was also observed in August and September of the preceding year.

### Drivers of coronavirus positivity

Results of the GLMM confirmed the significantly higher positivity of juveniles and immatures individuals and did not show any significant effect of species (*Hipposideros*, *Eonycteris* and *Pteropus,* the three most-captured species with at least one positive individual) or sex on CoV positivity (Table 1, Appendix Fig. 2).

**Table 1:** Results of the GLMM with the following references for the three explanatory variables: *E. spelaea* for species, female for sex and mature adults for age.

Variables	Odd ratios	p value	OR CI95
Intercept	0.014	< 10^−5^	0.00123–0.067
Species = H. larvatus	0.436	0.081	0.169–1.108
Species = P. lylei	0.117	0.053	0.007–0.929
Sex = male	1.566	0.209	0.778–3.18
Age = juveniles and immatures	3.245	0.005	1.462–7.758

*OR* odds ratio, *CI95* 95% confidence interval.

### Sequence analysis

**Figure 3:**
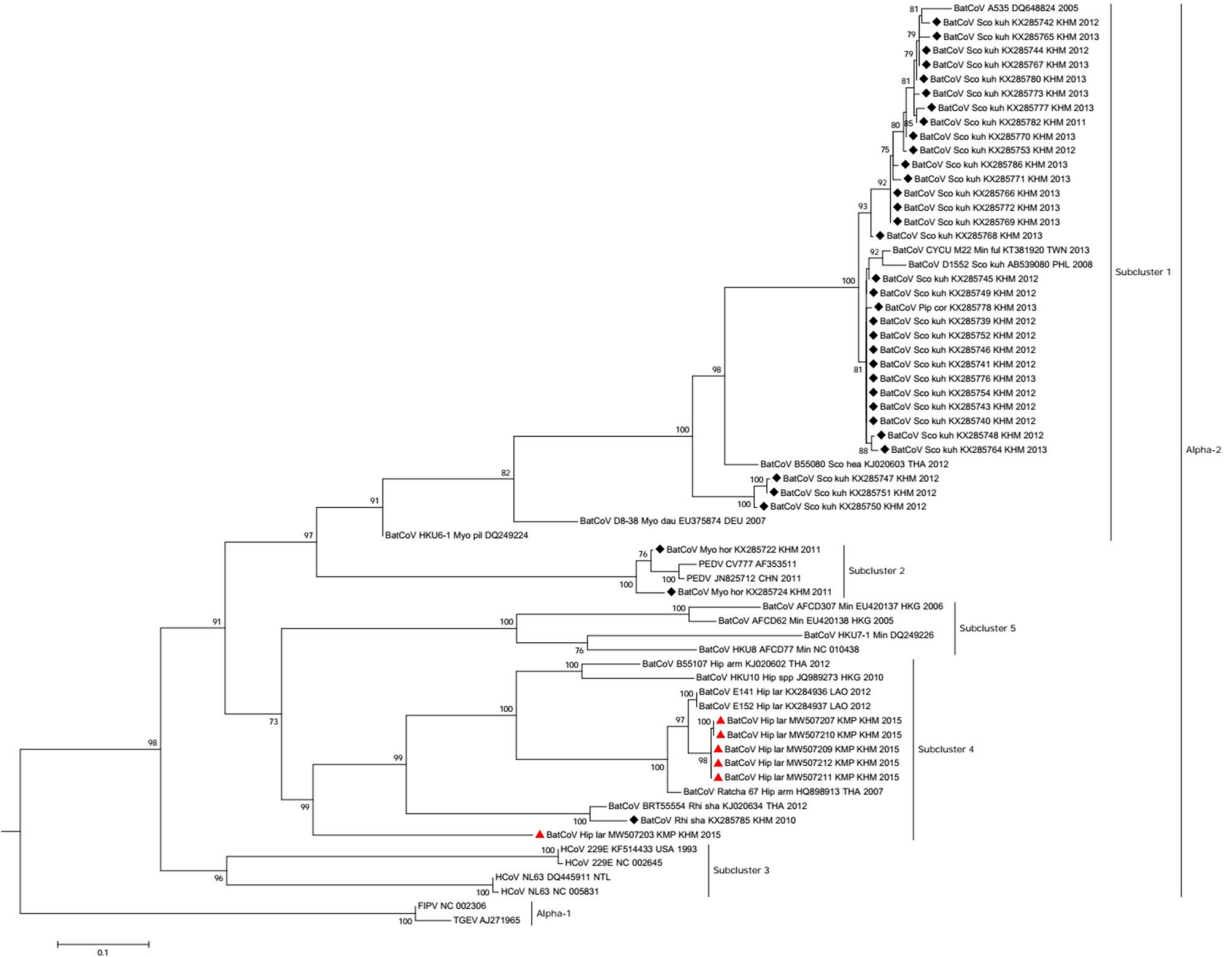

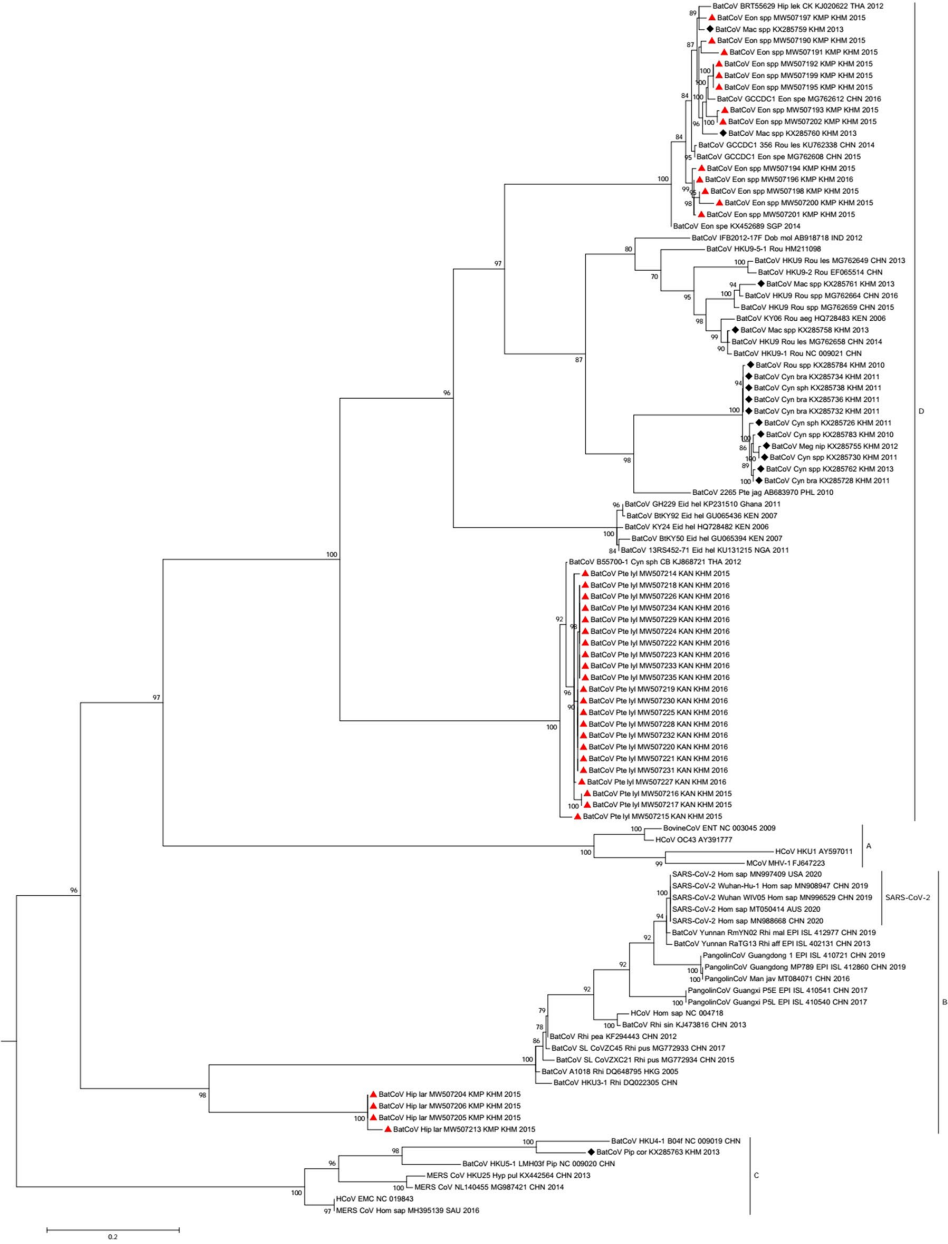
Phylogenetic tree of (**A**) alphaCoV and (**B**) betaCoV partial RdRp gene with modified primers. The sequences detected in Cambodia are marked by red triangles for strains in the current study and black diamonds for strains previously described by Lacroix et al.^6^. The tree was built using the maximum likelihood method based on the GTR + G4 + I model. The robustness of nodes was assessed with 1000 bootstrap replicates. Bootstrap values < 70 are not shown. The GenBank accession numbers of CoV detected in this study and included in the phylogenetic analysis are: MW507190–MW507207 and MW507209–MW507235.

Phylogenetic analysis using sequences (n = 45) detected with Watanabe-primers showed that CoVs detected in this study clustered in both alphacoronavirus and betacoronavirus groups (Fig. 3). CoVs detected in *H. larvatus* s.l. belonged to alphacoronavirus (n = 6) in subcluster 4 along with CoV detected in *Hipposideros spp.* from Laos, Thailand and Hong Kong. Another 4 CoVs sequences from *H. larvatus* s.l. clustered in a separate branch from other SARS-CoV related viruses within group B of betacoronavirus. All 13 CoV sequences from *E. spelaea* grouped with other bat CoVs detected in Asia (group D) including Cambodia, China, Thailand and Singapore. Interestingly, *P. lylei* (n = 22) harbored a unique and separate cluster with a CoV strain in *Cynopterus sphinx* from Thailand within betacoronavirus. Another phylogenetic tree using sequences generated with Quan-primers showed a similar clustering of Cambodian CoV in alpha and betacoronavirus (Appendix Fig. 1) and the additional CoV sequence from *H. larvatus* s.l. (MW507203) detected only with Quan-primers grouped with other sequences from the same bat species within alphacoronavirus.

### Diversity and reproductive phenology of ***Rhinolophus, Hipposideros, Eonycteris and Pteropus*** species

Existing literature indicates that at least 27 rhinolophid and 16 hipposiderid species are currently documented in mainland southeast China, Vietnam, Laos and Cambodia, including 19 and six (respectively) in mainland southeast China where SARS-CoV and SARS-CoV-2 are thought to have emerged in humans (Supplementary Materials). A single species of *Eonycteris* (*E. spelaea*) occurs throughout this region, whereas three *Pteropus* species (*P. lylei*, *P. vampyrus*, *P. hypomelanus*) occur in its southerly portions (Cambodia and south Vietnam).

**Figure 4:**
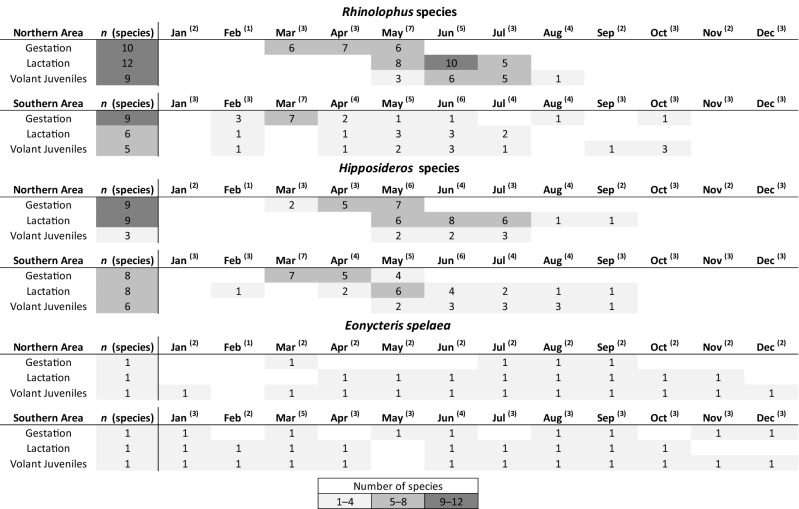
Summary of available data on the reproductive phenology of *Rhinolophus, Hipposideros and Eonycteris* species in mainland southeast China, Vietnam, Laos and Cambodia. The chart shows the number of species for which a reproduction phase has been observed in a given month of the year for the northern (above 16° N) and southern (below 16° N) portions of the region. Each cell is shaded proportionally according to the number of species for which data were retrieved during the literature review, whereas superscript figures refer to the number of studies for which reproductive data were available for the genus in a given month.

Our review of published and unpublished datasets (Supplementary Materials) reveals that basic information on reproductive phenology is lacking for most of these species and is summarized for the northern and southern portions of the region in Fig. 4. Overall, 16 studies and unpublished datasets including reproductive phenology data were obtained for rhinolophid bats (13 species), whereas 14 were obtained for hipposiderid bats (14 species) and four for *E. spelaea*. Aside from the present study, no data was available in the literature on the reproductive phenology of the three *Pteropus* species within our study region.

Though data are presently only available for a limited number of species, these indicate that the reproductive patterns of rhinolophid and hipposiderid bats in the northern zone (above 16° N) are characterized by restricted seasonal monoestry (one litter within a 2-month period each year), whereby gestation occurs from March to May and lactation largely from May to July, with species numbers for juveniles peaking in June–July. In contrast, the pattern is less clear in the southern zone where even fewer data are presently available, but nonetheless suggests that breeding is less seasonally constrained and may therefore qualify as extended seasonal monoestry (one litter per year within 2–7 months) or possibly even aseasonal monoestry (one litter at any time of the year) for some species. A rather different scenario applies to *E. spelaea,* whereby the species reproduces throughout the year. Within the northern zone, its reproductive patterns are characterized by seasonal bimodal polyoestry (two litters per year in close synchrony) with the majority of births occurring in March–April and August–September (and juveniles in May–July and September–November), whereas in the southern zone, the limited data currently available suggests that while births evidently occur throughout the year, these may peak in January and June–August.

## Discussion

Our longitudinal monitoring of several bat species in Cambodia suggests that prevalence of different CoVs is higher in juvenile and immature individuals than sexually mature bats of three species (*E. spelaea, H. larvatus s.l. and P. lylei*) in Cambodia. The same finding has been observed in other regions and this may be attributable to the greater susceptibility of these immunologically naive individuals compared to adults^13,17,18,21^. When reproduction is highly seasonal and synchronized within a species, the pulses of juveniles entering the general population can lead to sharp seasonal increases in pathogen circulation as observed for Marburg virus in *Rousettus aegyptiacus* in Uganda^14^. Reproduction phenology can thus shape the circulation pattern of a pathogen and influence its capacity to persist in a population^15,16^.

While our results suggest a higher circulation of CoVs in juvenile and immature individuals, the limitation of our sampling to 17 months does not allow us to assess potential seasonality of CoVs in these bat populations. Furthermore, our results also suggest some level of variability in the occurrence of a seasonal peak between years, with a peak observed in juveniles in June 2016 in *P. lylei* but an increased prevalence among immatures in September 2015. Similarly, the increased CoV prevalence observed between July and November 2015 in both *H. larvatus s.l.* and *E. spelaea* were not observed in September and November of the preceding year, although this may be due to limited sampling in 2014. These inter-annual differences may be associated with variations in population-level immunity which in turn impact the level of maternal immunity transferred to juveniles^40^. High levels of maternal immunity might thus explain a several-month delay in the peak of CoV circulation observed in young bats, a period corresponding to the waning of maternal antibodies^41^. Irrespectively, multiple consecutive years of monitoring would be needed to confirm these potential variations in a general seasonal circulation pattern of CoVs. Notwithstanding this, the association between CoV positivity and juvenile and immature individuals in our study suggests that surveillance of CoV could be targeted to the periods when juveniles and then sexually immature individuals form a higher proportion of bat populations.

The molecular characterization of CoVs detected during our study shows that several strains were circulating concomitantly in different bat species during the key period associated with an increased presence of young bats in the population. Hence, targeted sampling of coronaviruses during this period may help to survey the diversity of coronaviruses circulating in bat populations, including potentially zoonotic forms such as SARS-CoV-2 related viruses. Interestingly, bat species roosting in the same cave in Kampot Province did not share the same coronaviruses, which is consistent with previous associations between CoV clades and bat species observed in Cambodia^6^. As such, surveillance of CoVs should be conducted on all bat species present at a site, even when the different species roost together in close contact.

The primary message from our examination of the literature is that limited data are available on the reproductive phenology of rhinolophid and hipposiderid bats in southern China and Southeast Asia. More data should be collected to better understand the ecology of these potentially important species regarding the risk of future CoV emergences. Because these species and other insectivorous bats play important roles in Southeast Asian ecosystems and provide services that significantly benefit human populations in the region such as pest control and guano production^42,43^, improved knowledge of their ecology would also benefit public health and their conservation. Despite providing limited data, our literature review indicates that reproductive phenology is synchronized among *Rhinolophus* and *Hipposideros* species in the northern portion of the region (mainland southeast China, northern Laos and Vietnam above 16°N), with births peaking within a 2-month period in late spring (April–May). The same applies to many vespertilionid bat taxa that frequently share the same roost sites^37^ and this period corresponds with the start of the summer rains in the region^38,39^ when insect biomass increases markedly^44,45^, providing optimal conditions for the survival and development of young bats. In contrast, the breeding season appears to be longer in the southern portion of the region (below 16°N in southern Laos, southern Vietnam and Cambodia), although births of some rhinolophid and hipposiderid taxa and other insectivorous cave bats still appear to peak in April–May^26,43^. A similar situation may also apply nearer to the equator, for instance in Peninsular Malaysia (1°–6° N), where although some *Rhinolophus* species evidently breed all year round, the majority mostly produce young in the first half of the year, with parturition in several cave-roosting rhinolophids also confined to April–May^46,47^. More broadly, a pattern of late dry season pregnancies and wet season production of young is common for insectivorous bats in many parts of the tropics worldwide^48–50^, although it is also possible that the months of parturition could vary depending on the seasonality and modality of rainfall patterns. As such, these factors should be accounted for when planning surveillance of rhinolophid, hipposiderid and other cave roosting insectivorous bats.

To conclude, our study highlights the benefits of undertaking longitudinal monitoring of bat populations, including species hosting SARS-related CoVs. Ideally, such programs should be permanent to allow the collection of long-term data and observation of inter-annual trends that may reveal years with higher prevalence of viruses in bats and a subsequent higher risk of spillover to humans^51,52^. Although more robust data are needed, the association observed between CoV positivity and juvenile and immature bats could be used to guide surveillance and maximize the probability of detecting more strains of CoV. In southern China, Vietnam, Laos and Cambodia and potentially elsewhere in Southeast Asia, the period from June to November could be prioritized for CoV surveillance in young rhinolophid and hipposiderid bats if the objective is to maximize the probability of detecting the circulation of these potentially zoonotic strains. Although crude, this first level of targeted surveillance may be particularly relevant when the financial resources available to conduct such programs are limited. Similarly, additional data on bat reproductive phenology will help to refine the strategies for the surveillance of CoV in Southeast Asia, including the surveillance of SARS-CoV-2 related viruses in rhinolophid bats.

## Supplementary Information

Supplementary Information 1.

Supplementary Information 2.

Supplementary Information 3.

Supplementary Information 4.

Supplementary Information 5.

Supplementary Information 6.

Supplementary Information 7.
