# Supplementary figures and images for: Longitudinal monitoring in Cambodia suggests higher circulation of alpha and betacoronaviruses in juvenile and immature bats of three species
Source: Sci Rep. 2021 Dec 17;11:24145. doi: 10.1038/s41598-021-03169-z (PMC8683416; doi:10.1038/s41598-021-03169-z)

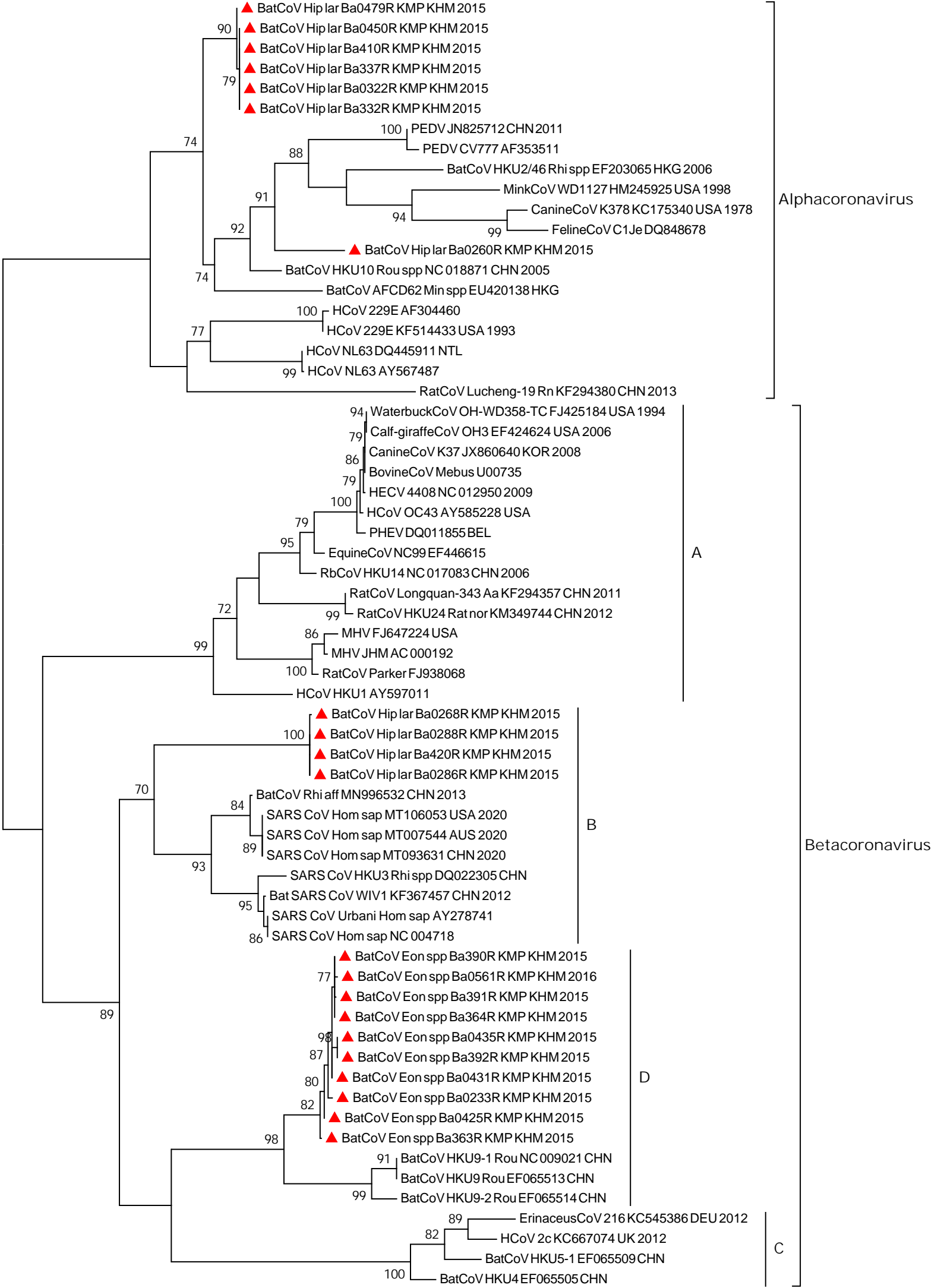

0.2

Supplement: Supplementary file 1 — Supplementary Information 1. [file 41598_2021_3169_MOESM1_ESM.pdf]

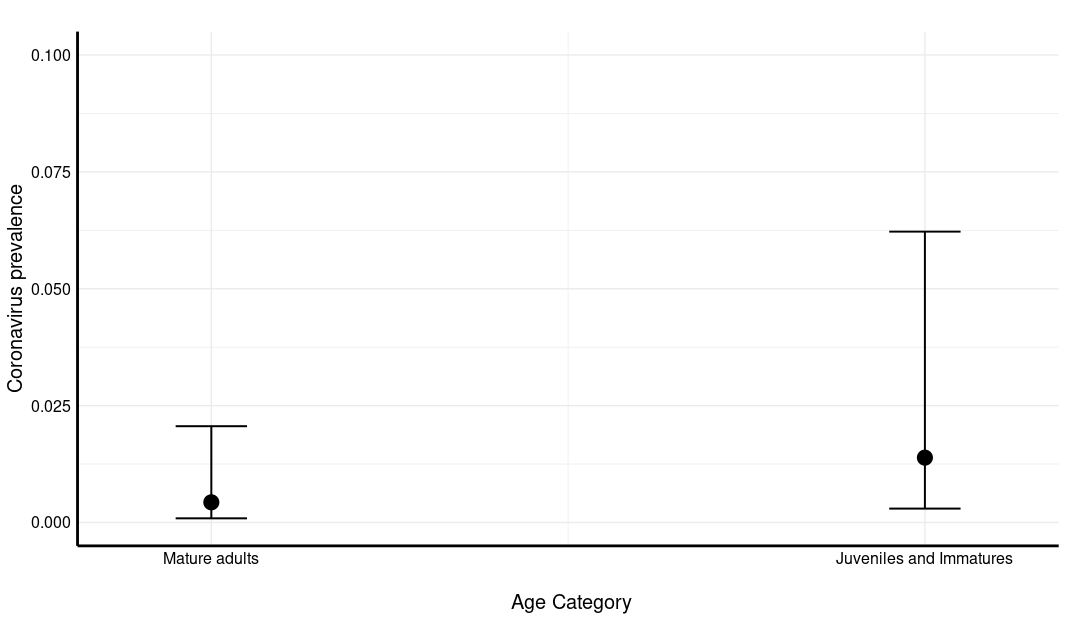

Supplement: Supplementary file 2 — Supplementary Information 2. [file 41598_2021_3169_MOESM2_ESM.jpeg]
