# Supplementary material for: Longitudinal monitoring in Cambodia suggests higher circulation of alpha and betacoronaviruses in juvenile and immature bats of three species
Source: Sci Rep. 2021 Dec 17;11:24145. doi: 10.1038/s41598-021-03169-z (PMC8683416; doi:10.1038/s41598-021-03169-z)
Supplement: Supplementary file 3 — Supplementary Information 3. [file 41598_2021_3169_MOESM3_ESM.docx]

Appendix Table 1: List of primer sequences adapted by Quan, PL et al., 2010 and Watanabe, S et al., 2010

| Primer’s name | Primer sequence | Amplicon size |
| --- | --- | --- |
| Quan, PL et al (2010). | | |
| Round 1: |  |  |
| CoV-FWD1 | CGTTGGIACWAAYBTVCCWYTICARBTRGG | 520 bp |
| CoV-RVS1 | GGTCATKATAGCRTCAVMASWWGCNACATG |  |
| Round 2: |  |  |
| CoV-FWD2 | GGCWCCWCCHGGNGARCAATT | 328 bp |
| CoV-RVS2 | GGWAWCCCCAYTGYTGWAYRTC |  |
| Modified from Watanabe, S *et al* (2010) | | |
| Round 1: |  |  |
| CoV-FWD3 | GGTTGGGAYTAYCCHAARTGTGA | 440 bp |
| CoV-RVS3 | CCATCATCASWYRAATCATCATA |  |
| Round 2: |  |  |
| CoV-FWD4/Ba | GAYTAYCCHAARTGTGAYAGAGC | 434 bp |
| CoV-RVS3 | Same reverse primer as round 1 |  |
