# Supplementary material for: Longitudinal monitoring in Cambodia suggests higher circulation of alpha and betacoronaviruses in juvenile and immature bats of three species
Source: Sci Rep. 2021 Dec 17;11:24145. doi: 10.1038/s41598-021-03169-z (PMC8683416; doi:10.1038/s41598-021-03169-z)
Supplement: Supplementary file 4 — Supplementary Information 4. [file 41598_2021_3169_MOESM4_ESM.docx]

**Appendix Table 2**: GenBank accession number of bat coronavirus using in the alignment.

| **No** | **Name of sequence** | **ID_Genbank** | **Strain** | **Host** | **Country** | **Year** | **Lineage** |
| --- | --- | --- | --- | --- | --- | --- | --- |
| 1 | BatCoV_D1552_Sco_kuh_AB539080_PHL_2008 | AB539080 | D1552 | Scotophilus kuhlii | Philippines | 2008 | Alphacoronavirus |
| 2 | BatCoV_2265_Pte_jag_AB683970_PHL_2010 | AB683970 | 2265 | Ptenochirus_jagori | Philippines | 2010 | Beltacoronavirus |
| 3 | BatCoV_IFB2012-17F_Dob_mol_AB918718_IND_2012 | AB918718 | IFB2012-17F | Dobsonia_moluccensis | India | 2012 | Beltacoronavirus |
| 4 | MHV_JHM_AC_000192 | AC000192 | JHM | Murine |  |  | Beltacoronavirus |
| 5 | HCoV_229E_AF304460 | AF304460 | 229E | Homo sapiens |  |  | Alphacoronavirus |
| 6 | PEDV_CV777_AF353511 | AF353511 | CV777 | Porcine |  |  | Alphacoronavirus |
| 7 | TGEV_AJ271965 | AJ271965 | PUR46-MAD | Pig | United State |  | Alphacoronavirus |
| 8 | HCoV_Hom_sap_AY278741_USA | AY278741 |  | Homo sapiens | United State |  | Beltacoronavirus |
| 9 | HCoV_OC43_AY391777 | AY391777 | OC43 | Homo sapiens |  |  | Beltacoronavirus |
| 10 | HCoV_NL63_AY567487 | AY567487 | Amsterdam I | Homo sapiens (NL63) |  |  | Alphacoronavirus |
| 11 | HCoV_OC43_AY585228_USA | AY585228 | OC43 | Homo sapiens | United State |  | Beltacoronavirus |
| 12 | HCoV_HKU1_AY597011 | AY597011 | HKU1 | Homo sapiens |  |  | Beltacoronavirus |
| 13 | PHEV_DQ011855_BEL | DQ011855 | VW572 | Porcine | Belgium |  | Beltacoronavirus |
| 14 | BatCoV_HKU3-1_Rhi_DQ022305_CHN | DQ022305 | HKU3-1 | Rhinolophus sp. (bat) | China |  | Beltacoronavirus |
| 15 | BatCoV_HKU6-1_Myo_pil_DQ249224 | DQ249224 | HKU6-1 | Myotis pilosus |  |  | Alphacoronavirus |
| 16 | BatCoV_HKU7-1_Min_DQ249226 | DQ249226 | HKU7-1 | Miniopterus |  |  | Alphacoronavirus |
| 17 | HCoV_NL63_DQ445911_NTL | DQ445911 | Amsterdam 057 | Homo sapiens (NL63) | Netherlands |  | Alphacoronavirus |
| 18 | BatCoV_A1018_Rhi_DQ648795_HKG_2005 | DQ648795 | A1018 | Rhinolophus sp. (bat) | Hong kong | 2005 | Beltacoronavirus |
| 19 | BatCoV_A535_DQ648824_2005 | DQ648824 | A535 | Bat |  | 2005 | Alphacoronavirus |
| 20 | FelineCoV_C1Je_DQ848678 | DQ848678 | C1Je | Feline |  |  | Alphacoronavirus |
| 21 | BatCoV_HKU9_Rou_EF065513_CHN | EF065513 | HKU9 | Rousettus sp. | China |  | Beltacoronavirus |
| 22 | BatCoV_HKU9-2_Rou_EF065514_CHN | EF065514 | HKU9-2 | Rousettus sp. | China |  | Beltacoronavirus |
| 23 | BatCoV_HKU2/46_Rhi_spp_EF203065_HKG_2006 | EF203065 | HKU2/46 | Rhinolophus sp. (bat) | Hong Kong | 2006 | Alphacoronavirus |
| 24 | Calf-giraffeCoV_OH3_EF424624_USA_2006 | EF424624 | OH3 | Calf-giraffe | United State | 2006 | Beltacoronavirus |
| 25 | EquineCoV_NC99_EF446615 | EF446615 | NC99 | Equine |  |  | Beltacoronavirus |
| 26 | BatCoV_D8-38_Myo_dau_EU375874_DEU_2007 | EU375874 | D8-38 | Myotis daubentonii | Germany | 2007 | Alphacoronavirus |
| 27 | BatCoV_AFCD307_Min_EU420137_HKG_2006 | EU420137 | AFCD307 | Miniopterus | Hong kong | 2006 | Alphacoronavirus |
| 28 | BatCoV_AFCD62_Min_EU420138_HKG_2005 | EU420138 | AFCD62 | Miniopterus | Hong kong | 2005 | Alphacoronavirus |
| 29 | WaterbuckCoV_OH-WD358-TC_FJ425184_USA_1994 | FJ425184 | OH-WD358-TC | Waterbuck | United State | 1994 | Beltacoronavirus |
| 30 | MCoV_MHV-1_FJ647223 | FJ647223 | MHV-1 | Murine |  |  | Beltacoronavirus |
| 31 | MHV_FJ647224_USA | FJ647224 |  | Murine | United State |  | Beltacoronavirus |
| 32 | RatCoV_Parker_FJ938068 | FJ938068 | Parker | Rat |  |  | Beltacoronavirus |
| 33 | BatCoV_BtKY50_Eid_hel_GU065394_KEN_2007 | GU065394 | BtKY50 | Eidolon_helvum | Kenya | 2007 | Beltacoronavirus |
| 34 | BatCoV_BtKY92_Eid_hel_GU065436_KEN_2007 | GU065436 | BtKY92 | Eidolon_helvum | Kenya | 2007 | Beltacoronavirus |
| 35 | BatCoV_HKU9-5-1_Rou_HM211098 | HM211098 | HKU9-5-1 | Rousettus spp |  |  | Beltacoronavirus |
| 36 | MinkCoV_WD1127_HM245925_USA_1998 | HM245925 | WD1127 | Mink | United State | 1998 | Alphacoronavirus |
| 37 | BatCoV_KY24_Eid_hel_HQ728482_KEN_2006 | HQ728482 | KY24 | Eidolon_helvum | Kenya | 2006 | Beltacoronavirus |
| 38 | BatCoV_KY06_Rou_aeg_HQ728483_KEN_2006 | HQ728483 | KY06 | Rousettus aegyptiacus | Kenya | 2006 | Beltacoronavirus |
| 39 | BatCoV_Ratcha_67_Hip_arm_HQ898913_THA_2007 | HQ898913 | Ratcha-67 | Hipposideros armiger | Thailand | 2007 | Alphacoronavirus |
| 40 | AvCoV_K561_Ana_cly_JN788871_HKG_2009 | JN788871 | K561/Anas clypeata | Anas clypeata | Hong kong | 2009 | Gammacoronavirus |
| 41 | PEDV_JN825712_CHN_2011 | JN825712 | BJ-2011-1 | Pig | China | 2011 | Alphacoronavirus |
| 42 | IBV_peafowl_JQ065042 | JQ065042 | peafowl (HKU15-44) | Pig | Hong kong | 2009 | Gammacoronavirus |
| 43 | PorCoV_HKU15-155_JQ065043_HKG_2010 | JQ065043 | HKU15-155 | Pig | Hong kong | 2010 | Deltacoronavirus |
| 44 | WECoV_HKU16-6847_WE_JQ065044_HKG_2007 | JQ065044 | HKU16-6847-WE | White-eye | Hong kong | 2007 | Deltacoronavirus |
| 45 | SpCoV_HKU17-6124_TrSp_JQ065045_HKG_2007 | JQ065045 | HKU17-6124-TrSp | Sparrow | Hong kong | 2007 | Deltacoronavirus |
| 46 | MRCoV_HKU18-chu3_OMR_JQ065046_HKG_2007 | JQ065046 | HKU18-chu3-OMR | Magpie-robin | Hong kong | 2007 | Deltacoronavirus |
| 47 | NHCoV_HKU19-6918_BICrNH_JQ065047_HKG_2007 | JQ065047 | HKU19-6918-BICrNH | Night-heron | Hong kong | 2007 | Deltacoronavirus |
| 48 | WiCoV_HKU20-9243_EuWi_JQ065048_HKG_2008 | JQ065048 | HKU20-9243-EuWi | Wigeon | Hong kong | 2008 | Deltacoronavirus |
| 49 | CMCoV_HKU21-8295_CM_JQ065049_HKG_2007 | JQ065049 | HKU21-8295-CM | Common moorhen | Hong kong | 2007 | Deltacoronavirus |
| 50 | BatCoV_HKU10_Hip_spp_JQ989273_HKG_2010 | JQ989273 | HKU10 | Hipposideros spp | Hong kong | 2010 | Alphacoronavirus |
| 51 | CanineCoV_K37_JX860640_KOR_2008 | JX860640 | K37 | Canine | South Korea | 2008 | Beltacoronavirus |
| 52 | CanineCoV_K378_KC175340_USA_1978 | KC175340 | K378 | Canine | United State | 1978 | Alphacoronavirus |
| 53 | ErinaceusCoV_216_KC545386_DEU_2012 | KC545386 | 216 | Erinaceus | Germany | 2012 | Beltacoronavirus |
| 54 | HCoV_2c_KC667074_UK_2012 | KC667074 | 2c | Homo sapiens | United Kingdom | 2012 | Beltacoronavirus |
| 55 | RatCoV_Longquan-343_Aa_KF294357_CHN_2011 | KF294357 | Longquan-343-Aa | Rat | China | 2011 | Beltacoronavirus |
| 56 | RatCoV_Lucheng-19_Rn_KF294380_CHN_2013 | KF294380 | Lucheng-19-Rn | Rat | China | 2013 | Alphacoronavirus |
| 57 | BatCoV_Rhi_pea_KF294443_CHN_2012 | KF294443 |  | Rhinolophus pearsonii | China | 2012 | Beltacoronavirus |
| 58 | BatCoV_WIV1_KF367457_CHN_2012 | KF367457 | WIV1 | Bat | China | 2012 | Beltacoronavirus |
| 59 | AvCoV_12WB63_KF437676_KOR_2012 | KF437676 | 12WB63 | Anas poecilorhyncha | South korea | 2012 | Gammacoronavirus |
| 60 | HCoV_229E_KF514433_USA_1993 | KF514433 | 229E | Homo sapiens | United State | 1993 | Alphacoronavirus |
| 61 | BatCoV_B55107_Hip_arm_KJ020602_THA_2012 | KJ020602 | B55107 | Hipposideros armiger | Thailand | 2012 | Alphacoronavirus |
| 62 | BatCoV_B55080_Sco_hea_KJ020603_THA_2012 | KJ020603 | B55080 | Scotophilus heathii | Thailand | 2012 | Alphacoronavirus |
| 63 | BatCoV_BRT55629_Hip_lek/CK_KJ020622_THA_2012 | KJ020622 | BRT55629 | Hipposideros_lekaguli | Thailand | 2012 | Beltacoronavirus |
| 64 | BatCoV_BRT55554_Rhi_sha_KJ020634_THA_2012 | KJ020634 | BRT55554 | Rhinolophus shameli | Thailand | 2012 | Alphacoronavirus |
| 65 | BatCoV_Rhi_sin_KJ473816_CHN_2013 | KJ473816 |  | Rhinolophus sinicus | China | 2013 | Beltacoronavirus |
| 66 | AvCoV_Ana_pla_KJ741881_USA_2009 | KJ741881 | OH38 | Anas platyrhynchos | United State | 2009 | Gammacoronavirus |
| 67 | BatCoV_B55700-1_Cyn_sph/CB_KJ868721_THA_2012 | KJ868721 | B55700-1 | Cynopterus_sphinx | Thailand | 2012 | Beltacoronavirus |
| 68 | AvCoV_S22_KM093879_MDG_2011 | KM093879 | S22 | Charadrius pecuarius | Madagascar | 2011 | Gammacoronavirus |
| 69 | RatCoV_HKU24_Rat_nor_KM349744_CHN_2012 | KM349744 | HKU24 | Rat | China | 2012 | Beltacoronavirus |
| 70 | BatCoV_GH229_Eid_hel_KP231510_Ghana_2011 | KP231510 | GH229 | Eidolon_helvum | Ghana | 2011 | Beltacoronavirus |
| 71 | DdCoV_DK_KT254275_CHN_2015 | KT254275 | DK | Duck | China | 2015 | Gammacoronavirus |
| 72 | BatCoV_CYCU_M22_Min_ful_KT381920_TWN_2013 | KT381920 | CYCU-M22 | Miniopterus fuliginosus | Taiwan | 2013 | Alphacoronavirus |
| 73 | BatCoV_13RS452-71_Eid_hel_KU131215_NGA_2011 | KU131215 | 13RS452-71 | Eidolon_helvum | Nigeria | 2011 | Beltacoronavirus |
| 74 | BatCoV_GCCDC1_356_Rou_les_KU762338_CHN_2014 | KU762338 | GCCDC1-356 | Rousettus leschenaulti | China | 2014 | Beltacoronavirus |
| 75 | BatCoV_E141_Hip_lar_KX284936_LAO_2012 | KX284936 | E141 | Hipposideros larvatus | Laos | 2012 | Alphacoronavirus |
| 76 | BatCoV_E152_Hip_lar_KX284937_LAO_2012 | KX284937 | E152 | Hipposideros larvatus | Laos | 2012 | Alphacoronavirus |
| 77 | BatCoV_Myo_hor_KX285722_KHM_2011 | KX285722 |  | Myotis horsfieldii | Cambodia | 2011 | Alphacoronavirus |
| 78 | BatCoV_Myo_hor_KX285724_KHM_2011 | KX285724 |  | Myotis horsfieldii | Cambodia | 2011 | Alphacoronavirus |
| 79 | BatCoV_Cyn_sph_KX285726_KHM_2011 | KX285726 |  | Cynopterus_sphinx | Cambodia | 2011 | Beltacoronavirus |
| 80 | BatCoV_Cyn_bra_KX285728_KHM_2011 | KX285728 |  | Cynopterus brachyotis | Cambodia | 2011 | Beltacoronavirus |
| 81 | BatCoV_Cyn_spp_KX285730_KHM_2011 | KX285730 |  | Cynopterus spp | Cambodia | 2011 | Beltacoronavirus |
| 82 | BatCoV_Cyn_bra_KX285732_KHM_2011 | KX285732 |  | Cynopterus brachyotis | Cambodia | 2011 | Beltacoronavirus |
| 83 | BatCoV_Cyn_bra_KX285734_KHM_2011 | KX285734 |  | Cynopterus brachyotis | Cambodia | 2011 | Beltacoronavirus |
| 84 | BatCoV_Cyn_bra_KX285736_KHM_2011 | KX285736 |  | Cynopterus brachyotis | Cambodia | 2011 | Beltacoronavirus |
| 85 | BatCoV_Cyn_sph_KX285738_KHM_2011 | KX285738 |  | Cynopterus_sphinx | Cambodia | 2011 | Beltacoronavirus |
| 86 | BatCoV_Sco_kuh_KX285739_KHM_2012 | KX285739 |  | Scotophilus kuhlii | Cambodia | 2012 | Alphacoronavirus |
| 87 | BatCoV_Sco_kuh_KX285740_KHM_2012 | KX285740 |  | Scotophilus kuhlii | Cambodia | 2012 | Alphacoronavirus |
| 88 | BatCoV_Sco_kuh_KX285741_KHM_2012 | KX285741 |  | Scotophilus kuhlii | Cambodia | 2012 | Alphacoronavirus |
| 89 | BatCoV_Sco_kuh_KX285742_KHM_2012 | KX285742 |  | Scotophilus kuhlii | Cambodia | 2012 | Alphacoronavirus |
| 90 | BatCoV_Sco_kuh_KX285743_KHM_2012 | KX285743 |  | Scotophilus kuhlii | Cambodia | 2012 | Alphacoronavirus |
| 91 | BatCoV_Sco_kuh_KX285744_KHM_2012 | KX285744 |  | Scotophilus kuhlii | Cambodia | 2012 | Alphacoronavirus |
| 92 | BatCoV_Sco_kuh_KX285745_KHM_2012 | KX285745 |  | Scotophilus kuhlii | Cambodia | 2012 | Alphacoronavirus |
| 93 | BatCoV_Sco_kuh_KX285746_KHM_2012 | KX285746 |  | Scotophilus kuhlii | Cambodia | 2012 | Alphacoronavirus |
| 94 | BatCoV_Sco_kuh_KX285747_KHM_2012 | KX285747 |  | Scotophilus kuhlii | Cambodia | 2012 | Alphacoronavirus |
| 95 | BatCoV_Sco_kuh_KX285748_KHM_2012 | KX285748 |  | Scotophilus kuhlii | Cambodia | 2012 | Alphacoronavirus |
| 96 | BatCoV_Sco_kuh_KX285749_KHM_2012 | KX285749 |  | Scotophilus kuhlii | Cambodia | 2012 | Alphacoronavirus |
| 97 | BatCoV_Sco_kuh_KX285750_KHM_2012 | KX285750 |  | Scotophilus kuhlii | Cambodia | 2012 | Alphacoronavirus |
| 98 | BatCoV_Sco_kuh_KX285751_KHM_2012 | KX285751 |  | Scotophilus kuhlii | Cambodia | 2012 | Alphacoronavirus |
| 99 | BatCoV_Sco_kuh_KX285752_KHM_2012 | KX285752 |  | Scotophilus kuhlii | Cambodia | 2012 | Alphacoronavirus |
| 100 | BatCoV_Sco_kuh_KX285753_KHM_2012 | KX285753 |  | Scotophilus kuhlii | Cambodia | 2012 | Alphacoronavirus |
| 101 | BatCoV_Sco_kuh_KX285754_KHM_2012 | KX285754 |  | Scotophilus kuhlii | Cambodia | 2012 | Alphacoronavirus |
| 102 | BatCoV_Meg_nip_KX285755_KHM_2012 | KX285755 |  | Megaerops niphanae | Cambodia | 2012 | Beltacoronavirus |
| 103 | BatCoV_Mac_spp_KX285758_KHM_2013 | KX285758 |  | Macroglossus sp | Cambodia | 2013 | Beltacoronavirus |
| 104 | BatCoV_Mac_spp_KX285759_KHM_2013 | KX285759 |  | Macroglossus sp | Cambodia | 2013 | Beltacoronavirus |
| 105 | BatCoV_Mac_spp_KX285760_KHM_2013 | KX285760 |  | Macroglossus sp | Cambodia | 2013 | Beltacoronavirus |
| 106 | BatCoV_Mac_spp_KX285761_KHM_2013 | KX285761 |  | Macroglossus sp | Cambodia | 2013 | Beltacoronavirus |
| 107 | BatCoV_Cyn_spp_KX285762_KHM_2013 | KX285762 |  | Cynopterus spp | Cambodia | 2013 | Beltacoronavirus |
| 108 | BatCoV_Pip_cor_KX285763_KHM_2013 | KX285763 |  | Pipistrellus coromandra | Cambodia | 2013 | Beltacoronavirus |
| 109 | BatCoV_Sco_kuh_KX285764_KHM_2013 | KX285764 |  | Scotophilus kuhlii | Cambodia | 2013 | Alphacoronavirus |
| 110 | BatCoV_Sco_kuh_KX285765_KHM_2013 | KX285765 |  | Scotophilus kuhlii | Cambodia | 2013 | Alphacoronavirus |
| 111 | BatCoV_Sco_kuh_KX285766_KHM_2013 | KX285766 |  | Scotophilus kuhlii | Cambodia | 2013 | Alphacoronavirus |
| 112 | BatCoV_Sco_kuh_KX285767_KHM_2013 | KX285767 |  | Scotophilus kuhlii | Cambodia | 2013 | Alphacoronavirus |
| 113 | BatCoV_Sco_kuh_KX285768_KHM_2013 | KX285768 |  | Scotophilus kuhlii | Cambodia | 2013 | Alphacoronavirus |
| 114 | BatCoV_Sco_kuh_KX285769_KHM_2013 | KX285769 |  | Scotophilus kuhlii | Cambodia | 2013 | Alphacoronavirus |
| 115 | BatCoV_Sco_kuh_KX285770_KHM_2013 | KX285770 |  | Scotophilus kuhlii | Cambodia | 2013 | Alphacoronavirus |
| 116 | BatCoV_Sco_kuh_KX285771_KHM_2013 | KX285771 |  | Scotophilus kuhlii | Cambodia | 2013 | Alphacoronavirus |
| 117 | BatCoV_Sco_kuh_KX285772_KHM_2013 | KX285772 |  | Scotophilus kuhlii | Cambodia | 2013 | Alphacoronavirus |
| 118 | BatCoV_Sco_kuh_KX285773_KHM_2013 | KX285773 |  | Scotophilus kuhlii | Cambodia | 2013 | Alphacoronavirus |
| 119 | BatCoV_Sco_kuh_KX285776_KHM_2013 | KX285776 |  | Scotophilus kuhlii | Cambodia | 2013 | Alphacoronavirus |
| 120 | BatCoV_Sco_kuh_KX285777_KHM_2013 | KX285777 |  | Scotophilus kuhlii | Cambodia | 2013 | Alphacoronavirus |
| 121 | BatCoV_Pip_cor_KX285778_KHM_2013 | KX285778 |  | Pipistrellus coromandra | Cambodia | 2013 | Alphacoronavirus |
| 122 | BatCoV_Sco_kuh_KX285780_KHM_2013 | KX285780 |  | Scotophilus kuhlii | Cambodia | 2013 | Alphacoronavirus |
| 123 | BatCoV_Sco_kuh_KX285782_KHM_2011 | KX285782 |  | Scotophilus kuhlii | Cambodia | 2011 | Alphacoronavirus |
| 124 | BatCoV_Cyn_spp_KX285783_KHM_2010 | KX285783 |  | Cynopterus spp | Cambodia | 2010 | Beltacoronavirus |
| 125 | BatCoV_Rou_spp_KX285784_KHM_2010 | KX285784 |  | Rousettus spp | Cambodia | 2010 | Beltacoronavirus |
| 126 | BatCoV_Rhi_sha_KX285785_KHM_2010 | KX285785 |  | Rhinolophus shameli | Cambodia | 2010 | Alphacoronavirus |
| 127 | BatCoV_Sco_kuh_KX285786_KHM_2013 | KX285786 |  | Scotophilus kuhlii | Cambodia | 2013 | Alphacoronavirus |
| 128 | MERS_CoV_HKU25_Hyp_pul_KX442564_CHN_2013 | KX442564 | HKU25 | Hyp | China | 2013 | Beltacoronavirus |
| 129 | BatCoV_Eon_spe_KX452689_SGP_2014 | KX452689 |  | Eonycteris_spelaea | Singapore | 2014 | Beltacoronavirus |
| 130 | BatCoV_GCCDC1_Eon_spe_MG762608_CHN_2015 | MG762608 | GCCDC1 | Eonycteris spelaea | China | 2015 | Beltacoronavirus |
| 131 | BatCoV_GCCDC1_Eon_spe_MG762612_CHN_2016 | MG762612 | GCCDC1 | Eonycteris spelaea | China | 2016 | Beltacoronavirus |
| 132 | BatCoV_HKU9_Rou_les_MG762649_CHN_2013 | MG762649 | HKU9 | Rousettus leschenaulti | China | 2013 | Beltacoronavirus |
| 133 | BatCoV_HKU9_Rou_les_MG762658_CHN_2014 | MG762658 | HKU9 | Rousettus leschenaulti | China | 2014 | Beltacoronavirus |
| 134 | BatCoV_HKU9_Rou_spp_MG762659_CHN_2015 | MG762659 | HKU9 | Rousettus spp | China | 2015 | Beltacoronavirus |
| 135 | BatCoV_HKU9_Rou_spp_MG762664_CHN_2016 | MG762664 | HKU9 | Rousettus spp | China | 2016 | Beltacoronavirus |
| 136 | AvCoV_Cal_ruf_MG764124_AUS_2017 | MG764124 |  | Calidris ruficollis | Australia | 2017 | Gammacoronavirus |
| 137 | MERS_CoV_NL140455_MG987421_CHN_2014 | MG987421 | NL140455 | Bat | China | 2014 | Beltacoronavirus |
| 138 | MERS_CoV_Hom_sap_MH395139_SAU_2016 | MH395139 |  | Homo sapiens | Saudi Arabia | 2016 | Beltacoronavirus |
| 139 | AvCoV_Mute_swan_MK617491_POL_2017 | MK617491 |  | Mute swan | Poland | 2017 | Gammacoronavirus |
| 140 | SARS-CoV-2_Hom_sap_MN988668_CHN_2020 | MN988668 |  | Homo sapiens | China | 2020 | Beltacoronavirus |
| 141 | BatCoV_Rhi_aff_MN996532_CHN_2013 | MN996532 |  | Rhinolophus affinis | China | 2013 | Beltacoronavirus |
| 142 | SARS-CoV-2_Hom_sap_MN997409_USA_2020 | MN997409 |  | Homo sapiens | United State | 2020 | Beltacoronavirus |
| 143 | SARS-CoV-2_Hom_sap_MT007544_AUS_2020 | MT007544 |  | Homo sapiens | Australia | 2020 | Beltacoronavirus |
| 144 | SARS-CoV-2_Hom_sap_MT050414_AUS_2020 | MT050414 |  | Homo sapiens | Australia | 2020 | Beltacoronavirus |
| 145 | PangolinCoV_MT084071_Man_jav_CHN_2016 | MT084071 |  | Manis javanica | China | 2016 | Beltacoronavirus |
| 146 | SARS-CoV-2_Hom_sap_MT093631_CHN_2020 | MT093631 |  | Homo sapiens | China | 2020 | Beltacoronavirus |
| 147 | SARS-CoV-2_Hom_sap_MT106053_USA_2020 | MT106053 |  | Homo sapiens | United State | 2020 | Beltacoronavirus |
| 148 | IBV_NC_001451 | NC_001451 | Beaudette | embryonated chicken eggs |  |  | Gammacoronavirus |
| 149 | FIPV_NC_002306 | NC_002306 | 79-1146 |  | United State |  | Alphacoronavirus |
| 150 | HCoV_229E_NC_002645 | NC_002645 | 229E | Homo sapiens |  |  | Alphacoronavirus |
| 151 | BovineCoV_ENT_NC_003045_2009 | NC_003045 | BCoV-ENT | Bovine |  | 2009 | Beltacoronavirus |
| 152 | HCoV_Hom_sap_NC_004718 | NC_004718 |  | Homo sapiens |  |  | Beltacoronavirus |
| 153 | HCoV_NL63_NC_005831 | NC_005831 | NL63 | Homo sapiens |  |  | Alphacoronavirus |
| 154 | BatCoV_HKU4-1_B04f_NC_009019_CHN | NC_009019 | HKU4-1-B04f | Bat | China |  | Beltacoronavirus |
| 155 | BatCoV_HKU5-1_LMH03f_Pip_NC_009020_CHN | NC_009020 | HKU5-1-LMH03f | Pipistrelle | China |  | Beltacoronavirus |
| 156 | BatCoV_HKU8_AFCD77_Min_NC_010438 | NC_010438 | HKU8-AFCD77 | Miniopterus |  |  | Alphacoronavirus |
| 157 | BWCoV_SW1_NC_010646 | NC_010646 | SW1 | Delphinapterus leucas (beluga whale) |  |  | Gammacoronavirus |
| 158 | TCoV_NC_010800 | NC_010800 | MG10 | Turkey |  |  | Gammacoronavirus |
| 159 | RbCoV_HKU14_NC_017083_CHN_2006 | NC_017083 | HKU14 | Rodent | China | 2006 | Beltacoronavirus |
| 160 | BatCoV_HKU10_Rou_spp_NC_018871_CHN_2005 | NC_018871 | HKU10 | Rousettus sp. | China | 2005 | Alphacoronavirus |
| 161 | HCoV_EMC_NC_019843 | NC_019843 | EMC | Homo sapiens |  |  | Beltacoronavirus |
| 162 | BovineCoV_Mebus_U00735 | U00735 | Mebus | Bovine |  |  | Beltacoronavirus |
| 163 | BatCoV_Eon_spp_MW507190_KMP_KHM_2015 | MW507190 | KMP15_Ba0233 | Eonycteris spp | Cambodia | 2015 | Beltacoronavirus |
| 164 | BatCoV_Hip_lar_MW507203_KMP_KHM_2015 | MW507203 | KMP15_Ba0260 | Hipposideros larvatus | Cambodia | 2015 | Alphacoronavirus |
| 165 | BatCoV_Hip_lar_MW507204_KMP_KHM_2015 | MW507204 | KMP15_Ba0268 | Hipposideros larvatus | Cambodia | 2015 | Beltacoronavirus |
| 166 | BatCoV_Hip_lar_MW507205_KMP_KHM_2015 | MW507205 | KMP15_Ba0286 | Hipposideros larvatus | Cambodia | 2015 | Beltacoronavirus |
| 167 | BatCoV_Hip_lar_MW507206_KMP_KHM_2015 | MW507206 | KMP15_Ba0288 | Hipposideros larvatus | Cambodia | 2015 | Beltacoronavirus |
| 168 | BatCoV_Hip_lar_MW507207_KMP_KHM_2015 | MW507207 | KMP15_Ba0322 | Hipposideros larvatus | Cambodia | 2015 | Alphacoronavirus |
| 169 | BatCoV_Eon_spp_MW507191_KMP_KHM_2015 | MW507191 | KMP15_Ba0425 | Eonycteris spp | Cambodia | 2015 | Beltacoronavirus |
| 170 | BatCoV_Eon_spp_MW507192_KMP_KHM_2015 | MW507192 | KMP15_Ba0431 | Eonycteris spp | Cambodia | 2015 | Beltacoronavirus |
| 171 | BatCoV_Eon_spp_MW507193_KMP_KHM_2015 | MW507193 | KMP15_Ba0435 | Eonycteris spp | Cambodia | 2015 | Beltacoronavirus |
| 172 | BatCoV_Eon_spp_MW507194_KMP_KHM_2015 | MW507194 | KMP15_Ba0438 | Eonycteris spp | Cambodia | 2015 | Beltacoronavirus |
| 173 | BatCoV_Hip_lar_MW507208_KMP_KHM_2015 | MW507208 | KMP15_Ba0450 | Hipposideros larvatus | Cambodia | 2015 | Alphacoronavirus |
| 174 | BatCoV_Hip_lar_MW507209_KMP_KHM_2015 | MW507209 | KMP15_Ba0479 | Hipposideros larvatus | Cambodia | 2015 | Alphacoronavirus |
| 175 | BatCoV_Eon_spp_MW507195_KMP_KHM_2015 | MW507195 | KMP15_Ba0495 | Eonycteris spp | Cambodia | 2015 | Beltacoronavirus |
| 176 | BatCoV_Eon_spp_MW507196_KMP_KHM_2016 | MW507196 | KMP15_Ba0561 | Eonycteris spp | Cambodia | 2016 | Beltacoronavirus |
| 177 | BatCoV_Hip_lar_MW507210_KMP_KHM_2015 | MW507210 | KMP15_Ba332 | Hipposideros larvatus | Cambodia | 2015 | Alphacoronavirus |
| 178 | BatCoV_Hip_lar_MW507211_KMP_KHM_2015 | MW507211 | KMP15_Ba337 | Hipposideros larvatus | Cambodia | 2015 | Alphacoronavirus |
| 179 | BatCoV_Eon_spp_MW507197_KMP_KHM_2015 | MW507197 | KMP15_Ba363 | Eonycteris spp | Cambodia | 2015 | Beltacoronavirus |
| 180 | BatCoV_Eon_spp_MW507198_KMP_KHM_2015 | MW507198 | KMP15_Ba364 | Eonycteris spp | Cambodia | 2015 | Beltacoronavirus |
| 181 | BatCoV_Eon_spp_MW507199_KMP_KHM_2015 | MW507199 | KMP15_Ba387 | Eonycteris spp | Cambodia | 2015 | Beltacoronavirus |
| 182 | BatCoV_Eon_spp_MW507200_KMP_KHM_2015 | MW507200 | KMP15_Ba390 | Eonycteris spp | Cambodia | 2015 | Beltacoronavirus |
| 183 | BatCoV_Eon_spp_MW507201_KMP_KHM_2015 | MW507201 | KMP15_Ba391 | Eonycteris spp | Cambodia | 2015 | Beltacoronavirus |
| 184 | BatCoV_Eon_spp_MW507202_KMP_KHM_2015 | MW507202 | KMP15_Ba392 | Eonycteris spp | Cambodia | 2015 | Beltacoronavirus |
| 185 | BatCoV_Hip_lar_MW507212_KMP_KHM_2015 | MW507212 | KMP15_Ba410 | Hipposideros larvatus | Cambodia | 2015 | Alphacoronavirus |
| 186 | BatCoV_Hip_lar_MW507213_KMP_KHM_2015 | MW507213 | KMP15_Ba420 | Hipposideros larvatus | Cambodia | 2015 | Beltacoronavirus |
| 187 | BatCoV_Pte_lyl_MW507214_KAN_KHM_2015 | MW507214 | KAN15_FF051 | Pteropus lylei | Cambodia | 2015 | Beltacoronavirus |
| 188 | BatCoV_Pte_lyl_MW507215_KAN_KHM_2015 | MW507215 | KAN15_FF058 | Pteropus lylei | Cambodia | 2015 | Beltacoronavirus |
| 189 | BatCoV_Pte_lyl_MW507216_KAN_KHM_2015 | MW507216 | KAN15_FF069 | Pteropus lylei | Cambodia | 2015 | Beltacoronavirus |
| 190 | BatCoV_Pte_lyl_MW507217_KAN_KHM_2015 | MW507217 | KAN15_FF070 | Pteropus lylei | Cambodia | 2015 | Beltacoronavirus |
| 191 | BatCoV_Pte_lyl_MW507218_KAN_KHM_2016 | MW507218 | KAN16_FF265 | Pteropus lylei | Cambodia | 2016 | Beltacoronavirus |
| 192 | BatCoV_Pte_lyl_MW507219_KAN_KHM_2016 | MW507219 | KAN16_FF290 | Pteropus lylei | Cambodia | 2016 | Beltacoronavirus |
| 193 | BatCoV_Pte_lyl_MW507220_KAN_KHM_2016 | MW507220 | KAN16_FF291 | Pteropus lylei | Cambodia | 2016 | Beltacoronavirus |
| 194 | BatCoV_Pte_lyl_MW507221_KAN_KHM_2016 | MW507221 | KAN16_FF294 | Pteropus lylei | Cambodia | 2016 | Beltacoronavirus |
| 195 | BatCoV_Pte_lyl_MW507222_KAN_KHM_2016 | MW507222 | KAN16_FF295 | Pteropus lylei | Cambodia | 2016 | Beltacoronavirus |
| 196 | BatCoV_Pte_lyl_MW507223_KAN_KHM_2016 | MW507223 | KAN16_FF299 | Pteropus lylei | Cambodia | 2016 | Beltacoronavirus |
| 197 | BatCoV_Pte_lyl_MW507224_KAN_KHM_2016 | MW507224 | KAN16_FF309 | Pteropus lylei | Cambodia | 2016 | Beltacoronavirus |
| 198 | BatCoV_Pte_lyl_MW507225_KAN_KHM_2016 | MW507225 | KAN16_FF311 | Pteropus lylei | Cambodia | 2016 | Beltacoronavirus |
| 199 | BatCoV_Pte_lyl_MW507226_KAN_KHM_2016 | MW507226 | KAN16_FF312 | Pteropus lylei | Cambodia | 2016 | Beltacoronavirus |
| 200 | BatCoV_Pte_lyl_MW507227_KAN_KHM_2016 | MW507227 | KAN16_FF313 | Pteropus lylei | Cambodia | 2016 | Beltacoronavirus |
| 201 | BatCoV_Pte_lyl_MW507228_KAN_KHM_2016 | MW507228 | KAN16_FF315 | Pteropus lylei | Cambodia | 2016 | Beltacoronavirus |
| 202 | BatCoV_Pte_lyl_MW507229_KAN_KHM_2016 | MW507229 | KAN16_FF316 | Pteropus lylei | Cambodia | 2016 | Beltacoronavirus |
| 203 | BatCoV_Pte_lyl_MW507230_KAN_KHM_2016 | MW507230 | KAN16_FF317 | Pteropus lylei | Cambodia | 2016 | Beltacoronavirus |
| 204 | BatCoV_Pte_lyl_MW507231_KAN_KHM_2016 | MW507231 | KAN16_FF319 | Pteropus lylei | Cambodia | 2016 | Beltacoronavirus |
| 205 | BatCoV_Pte_lyl_MW507232_KAN_KHM_2016 | MW507232 | KAN16_FF323 | Pteropus lylei | Cambodia | 2016 | Beltacoronavirus |
| 206 | BatCoV_Pte_lyl_MW507233_KAN_KHM_2016 | MW507233 | KAN16_FF326 | Pteropus lylei | Cambodia | 2016 | Beltacoronavirus |
| 207 | BatCoV_Pte_lyl_MW507234_KAN_KHM_2016 | MW507234 | KAN16_FF328 | Pteropus lylei | Cambodia | 2016 | Beltacoronavirus |
| 208 | BatCoV_Pte_lyl_MW507235_KAN_KHM_2016 | MW507235 | KAN16_FF329 | Pteropus lylei | Cambodia | 2016 | Beltacoronavirus |
| 209 | SARS-CoV-2_Wuhan-Hu-1_Hom_sap_MN908947_CHN_2019 | MN908947 | Wuhan-Hu-1 | Homo sapiens | China | 2019 | Beltacoronavirus |
| 210 | SARS-CoV-2_Wuhan/WIV05_Hom_sap_MN996529_CHN_2019 | MN996529 | Wuhan/WIV05 | Homo sapiens | China | 2019 | Beltacoronavirus |
| 211 | BatCoV_Yunnan/RmYN02_Rhi_mal_EPI_ISL_412977_CHN_2019 | EPI_ISL_412977 | Yunnan/RmYN02 | Rhinolophus malayanus | China | 2019 | Beltacoronavirus |
| 212 | BatCoV_Yunnan/RaTG13_Rhi_aff_EPI_ISL_402131_CHN_2013 | EPI_ISL_402131 | Yunnan/RaTG13 | Rhinolophus affinis | China | 2013 | Beltacoronavirus |
| 213 | PangolinCoV_Guangdong/MP789_EPI_ISL_412860_CHN_2019 | EPI_ISL_412860 | Guangdong/MP789 | Pangolin | China | 2019 | Beltacoronavirus |
| 214 | PangolinCoV_Guangdong/1_EPI_ISL_410721_CHN_2019 | EPI_ISL_410721 | Guangdong/1 | Pangolin | China | 2019 | Beltacoronavirus |
| 215 | PangolinCoV_Guangxi/P5L_EPI_ISL_410540_CHN_2017 | EPI_ISL_410540 | Guangxi/P5L | Pangolin | China | 2017 | Beltacoronavirus |
| 216 | PangolinCoV_Guangxi/P5E_EPI_ISL_410541_CHN_2017 | EPI_ISL_410541 | Guangxi/P5E | Pangolin | China | 2017 | Beltacoronavirus |
| 217 | BatCoV_SL_CoVZC45_Rhi_pus_MG772933_CHN_2017 | MG772933 | SL_CoVZC45 | Rhinolophus pusillus | China | 2017 | Beltacoronavirus |
| 218 | BatCoV_SL_CoVZXC21_Rhi_pus_MG772934_CHN_2015 | MG772934 | SL_CoVZXC21 | Rhinolophus pusillus | China | 2015 | Beltacoronavirus |
