# Supplementary material for: Longitudinal monitoring in Cambodia suggests higher circulation of alpha and betacoronaviruses in juvenile and immature bats of three species
Source: Sci Rep. 2021 Dec 17;11:24145. doi: 10.1038/s41598-021-03169-z (PMC8683416; doi:10.1038/s41598-021-03169-z)
Supplement: Supplementary file 7 — Supplementary Information 7. [file 41598_2021_3169_MOESM7_ESM.docx]

**Appendix Table 1.** List of primer sequences adapted by Quan, PL et al., 2010 and Watanabe, S et al., 2010

**Appendix Table 2.** GenBank accession number of bat coronavirus using in the alignment.

**Appendix Table 3.** Results from the coronavirus PCR by species and reproductive status.

**Appendix Table 4.** Detailed results from the coronavirus PCR by species, reproductive status and sampling session.

**Appendix Figure 1.** Phylogenetic tree of CoV partial RdRp gene generated with Quan primers. The Cambodian sequences in this study are marked by red triangle. The tree was built using the maximum likelihood method based on the GTR+G4+I model. The robustness of nodes was assessed with 1000 bootstrap replicates. Bootstrap values <60 are not shown. The GenBank accession numbers of CoV detected in this study and included in the phylogenetic analysis are: MW507190 - MW507207 and MW507209 - MW507235.

**Appendix Figure 2.** Probability of coronavirus positivity per Age category.
